# Long-term Local Control Following CEA-targeted Fluorescence-guided Surgery in Patients With Locally Advanced and Recurrent Rectal Cancer

**DOI:** 10.1007/s11307-025-02021-4

**Published:** 2025-06-05

**Authors:** Mats I. Warmerdam, Davy M. J. Creemers, Miranda Kusters, Koen C. M. J. Peeters, Fabian A. Holman, J. Sven D. Mieog, Francoise Cailler, Pim J. W. A. Burger, Jacobus Burggraaf, Harm J. T. Rutten, Cornelis Verhoef, Alexander L. Vahrmeijer, Denise E. Hilling

**Affiliations:** 1 Department of Surgery, Leiden University Medical Center, Leiden, The Netherlands; 2 Department of Surgery, Catharina Hospital Eindhoven, Eindhoven, The Netherlands; 3 Department of GROW, School for Developmental Biology & Oncology, Maastricht University, Maastricht, the Netherlands; 4 Department of Surgery, Amsterdam University Medical Center (AUMC), Amsterdam, The Netherlands; 5 Surgimab, Montpellier, France; 6 Center for Human Drug Research (CHDR), Leiden, The Netherlands; 7 Department of Surgical Oncology and Gastrointestinal Surgery, Erasmus MC Cancer Institute, Rotterdam University Medical Center, Rotterdam, The Netherlands

**Keywords:** Colorectal neoplasia, Optical imaging, Near-infrared guided surgery, Fluorescence, CEA-targeted imaging, Survival, Follow-up

## Abstract

**Purpose:**

In our previous phase 2 trial, patients with locally advanced (LARC) or locally recurrent rectal cancer (LRRC) received SGM-101, a CEA-targeted fluorescent agent, to enable real-time near-infrared fluorescence (NIRF) guided surgery. This study demonstrated that SGM-101 enabled additional tumor removal in some patients and supported less invasive surgery in others. Despite this positive intraoperative effect, the impact on long-term tumor control is unknown. Therefore, in this article we report the long-term outcomes of all rectal cancer patients that participated to the trial.

**Procedures:**

For all 29 LARC and LRRC patients that participated in the SGM-101 phase 2 trial, follow-up data were collected. Main outcome measure was 5-year local tumor control.

**Results:**

The median follow-up of all patients was 5.0 years (IQR 4.5–5.5). Of the 12 LARC patients, three (25%) patients developed a local recurrence. The two patients in whom NIRF-guided surgery resulted in less invasive surgery remained locally recurrence-free. Among the 17 patients undergoing curative surgery for LRRC, 11 (65%) patients developed a local re-recurrence. Of the three patients who had an R0 instead of R1 as a direct result of SGM-101 guided surgery, one patient developed a local re-recurrence (33%), while the other two remained local recurrence-free.

**Conclusions:**

This is the first study to report follow-up data on patients undergoing tumor-targeted NIRF-guided surgery. Although SGM-101 resulted in warranted changes in surgical management intra-operatively, no improved long-term benefit could be observed for the entire cohort. However, the subset of patients whose surgical approach was modified based on NIRF – either by performing less invasive surgery or removing additional malignant tissue—showed favorable long-term outcomes. Results from ongoing large trials are awaited.

**Supplementary Information:**

The online version contains supplementary material available at 10.1007/s11307-025-02021-4.

## Introduction

In locally advanced rectal cancer (LARC) and locally recurrent rectal cancer (LRRC) surgery, surgeons commonly encounter challenges in achieving complete tumor resections (R0). Amongst other things, this can be attributed to the intricate anatomy of the small pelvis, fibrosis caused by neoadjuvant chemoradiotherapy, and in the case of LRRC, altered anatomy as a result of previous total mesorectal excision (TME) surgery. Consequently, the R1 ratio is 10–20% in LARC and approximately 45% in LRRC, with certain subgroups reaching levels as high as 72% [1–5]. For both LARC and LRRC tumor positive resection margins (R1) are associated with increased local recurrence rates and decreased overall survival [1, 2, 4, 6–8]. These, local (re-)recurrences are accompanied by high morbidity such as pain, gastro-intestinal symptoms, urinary and sexual dysfunction and lower limb musculoskeletal pain and weakness [9]. Thus, obtaining complete tumor removal is of the utmost importance.

Near-infrared fluorescence (NIRF) guided surgery is a technique that can be used to visualize tumor tissue [10]. It involves the administration of a fluorescent agent that selectively accumulates within or around the tumor. Subsequently the signal can be detected and displayed in real-time by a dedicated NIRF camera system [10].

**Fig. 1:**
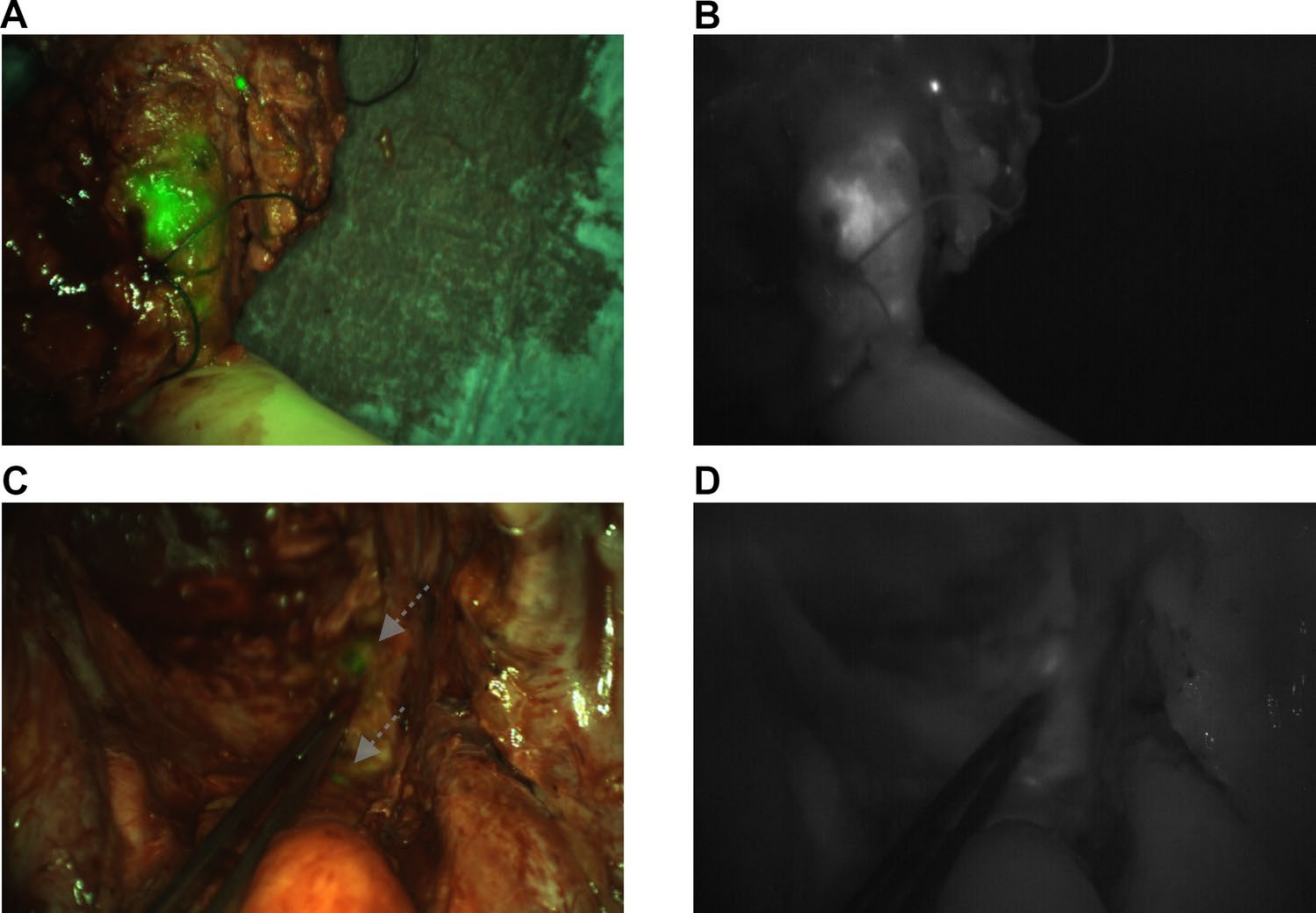
Intra-operative fluorescence imaging after resection of locally recurrent rectal cancer (LRRC) clinically assessed as clear margins (R0), but SGM-101 imaging revealed positive margins (R1). **A** RGB-image with fluorescent overlay showing LRRC-specimen expressing strong fluorescence at the resection margin, suggestive of positive margins (R1). **B** Corresponding monochromatic fluorescent image. **C** Two remaining small fluorescent hotspots confirmed as malignant after re-resection. **D** Corresponding monochromatic fluorescent image

**Fig. 2:**
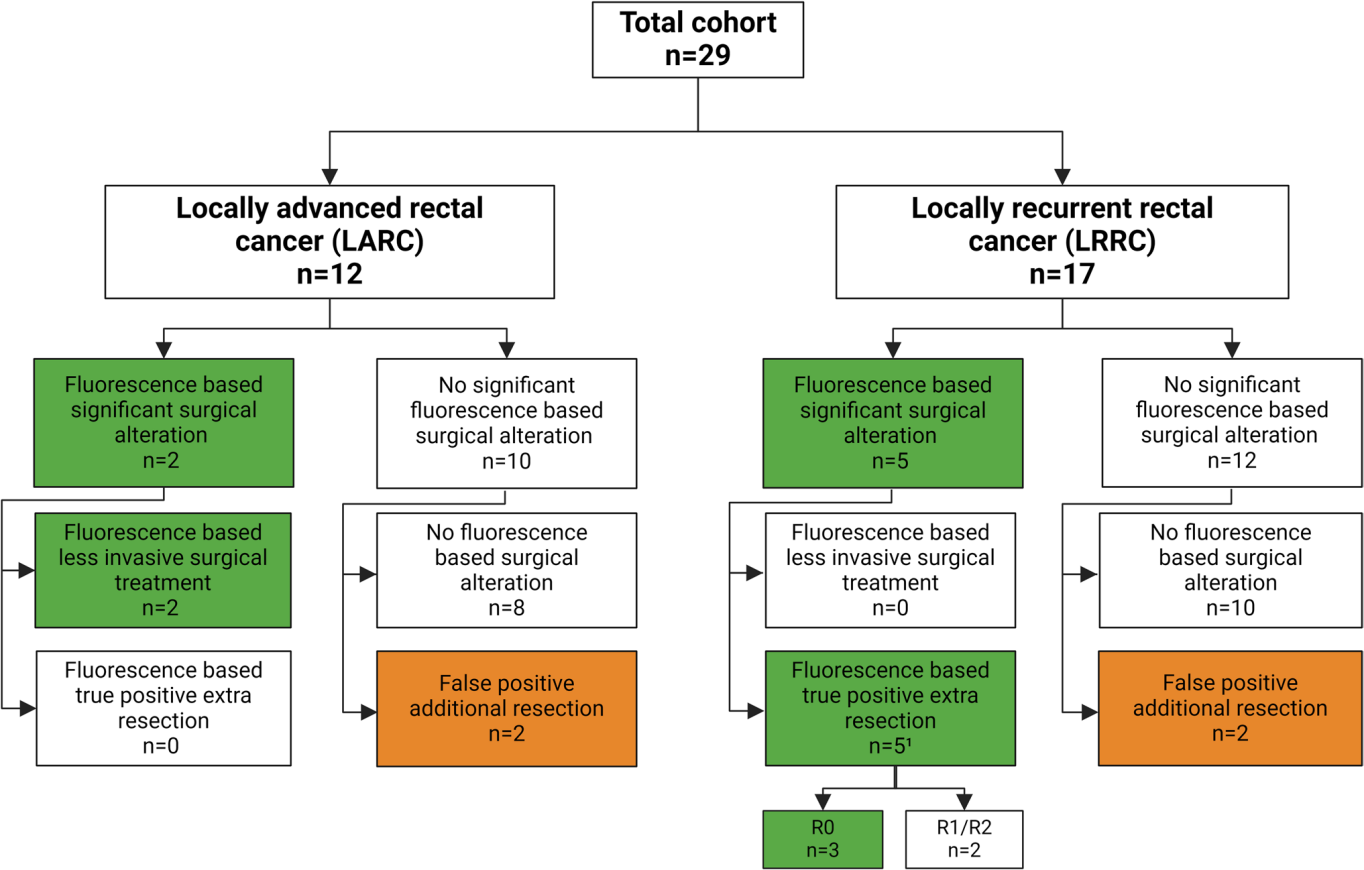
Overview SGM-101 phase-2 cohort and alterations in surgical plan, divided by type of cancer (LARC or LRRC), significant or no significant alteration in surgical plan and the type of surgical alteration. Patients were classified as having a "significant alteration based on fluorescence-guided surgery" if their surgical plan was exclusively modified due to NIRF and resulted in a substantial outcome, defined as: 1) removal of additional malignant tissue; or 2) less invasive surgery. All fluorescence-guided false positive additional resections (fluorescent, benign) were minor in size and did not lead to post-operative morbidity higher than expected. ^1^ One patient had both a false positive additional resection as two true positive additional resections

Recently, we published results of a phase 2 clinical trial that was conducted from 2016–2018 involving 29 patients undergoing elective surgery for LARC or LRRC with NIRF using SGM-101 [11, 12]. The investigational drug SGM-101 consists of the fluorophore BM-104 covalently bound to a chimeric monoclonal antibody targeting carcinoembryonic antigen (CEA). CEA is a well-known tumor marker, overexpressed in colorectal cancer [13]. The trial showed that intra-operative use of SGM-101 led to significant alterations in the surgical plan for 7 out of 29 patients. In five of these cases (all LRRC), SGM-101 fluorescence revealed residual malignant tissue in the pelvic cavity that would have otherwise remained undetected. The consequent additional resections were confirmed malignant by pathology and improved margins from R1 to R0 in three of the patients (See Fig. 1 for illustration). In two LARC patients, the *absence* of fluorescence allowed for less invasive surgery than initially planned [11, 12]. Despite the significant alterations in these seven patients, in five patients additional small-sized false positive resections were performed (fluorescent, not malignant) [11, 12]. An overview of the above findings is summarized in Fig. 2. A more detailed description per case can be found in Table 1 of the supplementary file.

Despite an increasing number of successful tumor-targeted NIRF phase 2/3 clinical trials that show intra-operative benefit, up to our knowledge none of them have assessed the long-term benefit for patients so far. Therefore, the aim of this study is to assess the long-term impact on rectal cancer patients who underwent tumor-targeted NIRF-guided surgery using SGM-101 as part of the phase 2 trial.

## Patients and Methods

### Patients

All patients that underwent resection of LARC or LRRC with SGM-101 (SurgiMab, Montpellier, France) as part of the phase 2 clinical trial at the Leiden University Medical Center (LUMC) or Catharina Hospital Eindhoven (CZE) between January 2016 and June 2018 were included in the analysis (NCT02973672) [11, 12]. Participating patients received dosages of 5–15 mg, 2–6 days before surgery. The protocol was approved by the institutional review board and participants provided written informed consent. The study design and patient selection have been described in detail previously [11, 12]. Conforming to Dutch guidelines, follow-up of these patients involved abdominal and thoracic CT imaging and blood CEA measurements at set times, in all patients.

### Data Collection and Definitions

Patient characteristics, surgical outcome (R0/R1), change of surgical plan due to NIRF and pathology results were already available as this data was collected during our phase 2 trial. For this study additional parameters and follow-up data from the electronic health record (EHR) were collected: local (re-)recurrence during the follow-up; time to local (re-)recurrence in months from surgery to its first visualization on CT-scan, MRI-scan or PET-CT-scan; manifestation of metastatic lesions; time to metastases in months from surgery to first visualization on CT-scan, MRI-scan or PET-CT-scan; mortality; disease-related mortality; time to disease-related mortality in months. Additionally, the variable ‘response to neoadjuvant therapy’ was collected from the pathology report. In cases where this information was not reported, the response rate from the pre-operative re-staging MRI was used.

A complete (R0) resection was defined as a resection margin > 1 mm in LARC patients [14] and > 0 mm in LRRC patients [15]. LARC was defined as a rectal or rectosigmoid T3/T4 tumor with involved mesorectal fascia on pre-neoadjuvant radiologic assessment. LRRC was defined as a local recurrence of a rectal, rectosigmoid or distal sigmoid cancer in the pelvic area. Patients are classified as having a “significant alteration based on NIRF” when their surgical plan was modified exclusively due to NIRF and resulted in a substantial different outcome, defined as: 1) removal of additional malignant tissue (true positive; fluorescent and malignant, while clinically not suspect) or 2) less invasive surgical treatment, defined as treatment that is less extensive than initially planned because of the absence of fluorescence at a previously suspected area. Patients were categorized as “no significant alteration based on NIRF”  when NIRF had either no impact on the surgical plan or when any NIRF-related additional resection was false positive (fluorescent but benign tissue).

### Statistical Analysis

All analyses were performed using the Statistical Package for Social Sciences (SPSS, IBM Corporation, Armonk, NY, USA). In this study continuous data are presented as median with interquartile range, while categorical data are reported as count with percentage. For continuous data with only three values or less, the median is presented alongside the corresponding values enclosed within brackets. Overall survival and local recurrence-free survival were calculated by the method of Kaplan–Meier.

## Results

For all 29 LARC and LRRC patients from the phase 2 trial, follow-up data until August 3rd 2023 could be obtained, encompassing 12 LARC and 17 LRRC patients. Median follow-up until last oncologic assessment for patients that were alive at the time of analysis was 5.0 years (IQR 4.5–5.5). Baseline patient characteristics, tumor characteristics, local (re-)recurrence rates and survival outcomes are summarized in Table 1. Among LARC patients, the local recurrence rate was 25% (3/12), 58% (7/12) developed metastases and 33% (4/12) deceased related to the disease. One patient deceased 2 months after surgery due to a non-disease related cause. In the LRRC cohort, 65% of patients (11/17) were diagnosed with a local re-recurrence, 71% (12/17) developed metastases and 53% (9/12) deceased related to the disease.

**Table 1:** Demographics, pre/peri-operative characteristics, pathology and 5-year follow-up of 29 patients who underwent SGM-101 guided rectal cancer surgery from 2016–2018

	Patient characteristics, pathology and follow-up results			
		LARC	LRRC	Total
	Total, *n*	12	17	29
Patient characteristics	Age at time of operation, y, med (IQR)	65 (59—69)	59 (55—65)	62 (57—68)
	Women, *n* (%))	5 (42)	7 (41)	12 (41)
	ASA-score, med (IQR)	2 (2–3)	2 (2–2)	2 (2–2)
	Previous surgery for LRRC, *n* (%)	NA	1 (6%)	NA
Treatment characteristics	Neoadjuvant therapy, *n* (%))	12 (100)	17 (100)	29 (100)
	IORT, *n* (%)	4 (33)	17 (100)	21 (72)
NIRF-characteristics	Surgical plan alteration due to NIRF, *n* (%)	4 (33)	7 (41)	11 (38)
	True positive additional resection, *n* (%)	0 (0)	5 (29)^1^	5 (29)
	False positive additional resection, *n* (%)	2 (8)	3 (17)^1^	5 (17)
	Less invasive surgical treatment, *n* (%)	2 (17)	0 (0)	2 (7)
Pathology outcome	Response to neoadjuvant therapy^2^ No response, *n* (%) Partial response, *n* (%) Complete response, *n* (%)	1 (8) 7 (58) 4 (33)	10 (59) 3 (18) 4 (26)	11 (38) 10 (35) 8 (28)
	pT stage, *n *(%) T0, *n* (%) (complete response) T1, *n* (%) T2, *n* (%) T3, *n* (%) T4, *n* (%)	4 (33) 1 (8) 2 (17) 5 (42) 0 (0)	NA NA NA NA NA	NA NA NA NA NA
	pN stage, *n* (%) N0, *n* (%) N1, *n* (%) N2, *n* (%)	9 (75) 2 (17) 1 (8)	NA NA NA	NA NA NA
	Mucinous, *n* (%)	1 (8)	2 (12)	3 (10)
	R0, *n* (%)	12 (100)	13 (77)	25 (86)
Follow-up	local (re-)recurrence, *n* (%)	3 (25)	11 (65)	14 (48)
	Time to local (re-)recurrence in months, med (IQR)	8 (8;11;45)	14 (5–24)	14 (6–28)
	Multifocal, *n* (% of local recurrences)	1 (33)	6 (55)	7 (50)
	Metastases, *n* (%)	7 (58)	12 (71)	19 (66)
	Time to metastases in months, med (IQRs)	7 (5–29)	7 (4–16)	7 (5–17)
	Metastases location, Liver, *n* (%) Lung, *n* (%) Peritoneum, *n* (%) Distant lymph nodes, *n* (%) Central nervous system, *n* (%) Other, *n* (%)	6 (50) 3 (25) 0 (0) 1 (8) 0 (0) 0 (0)	2 (12) 8 (47) 5 (29) 5 (29) 2 (12) 2 (12)	8 (28) 11 (38) 5 (17) 6 (21) 2 (7) 2 (7)
	Mortality (disease related), *n* (%)	4 (33)	9 (53)	13 (45)
	Time to death in months (disease related), med (IQR)	47 (28–58)	34 (30–40)	35 (30–53)
	Mortality (non-disease related), *n* (%)	2 (0)	0 (0)	2 (6.8)
	Time to death in months (non-disease related), med (IQR)	30 (2;58)	N.A	30 (2;58)

*LRRC* locally recurrent rectal cancer, *LARC* locally advanced rectal cancer, *NIRF* near-infrared fluorescence, *IORT* intraoperative radiotherapy, R0 = margins negative for tumor cells, *R1* margins positive for tumor cells, *N.A.* not applicable

True positive NIRF-guided resectio*n = *additional resection performed during surgery solely based on fluorescence, that turned out to be malignant at pathology assessment

False positive NIRF-guided resectio*n = *additional resection performed during surgery solely based on fluorescence, that turned out to be benign at pathology assessment

NIRF-guided less invasive surgical treatment = less extensive surgical treatment than initially planned because of the absence of fluorescence at a previously suspected area

For continuous data with only 3 values or less, the median is presented alongside the corresponding values enclosed within brackets

^1^ One patient had both a false positive additional resection as two true positive additional resections

^2^ In nine patients, data was missing for the variable ‘response to neoadjuvant therapy’ at histology assessment, for these cases the response rate from the pre-operative re-staging MRI was used

Overall 5-year local (re-)recurrence-free survival was 35% (95% CI 4,8–65,2) for LARC patients and 24% (95% CI 3,3–43,7) for LRRC patients, while overall 5-year survival was 50% (95% CI 21,8–78,2) and 53% (95% CI 29,2–76,6) for LARC and LRRC respectively.

**Fig. 3:**
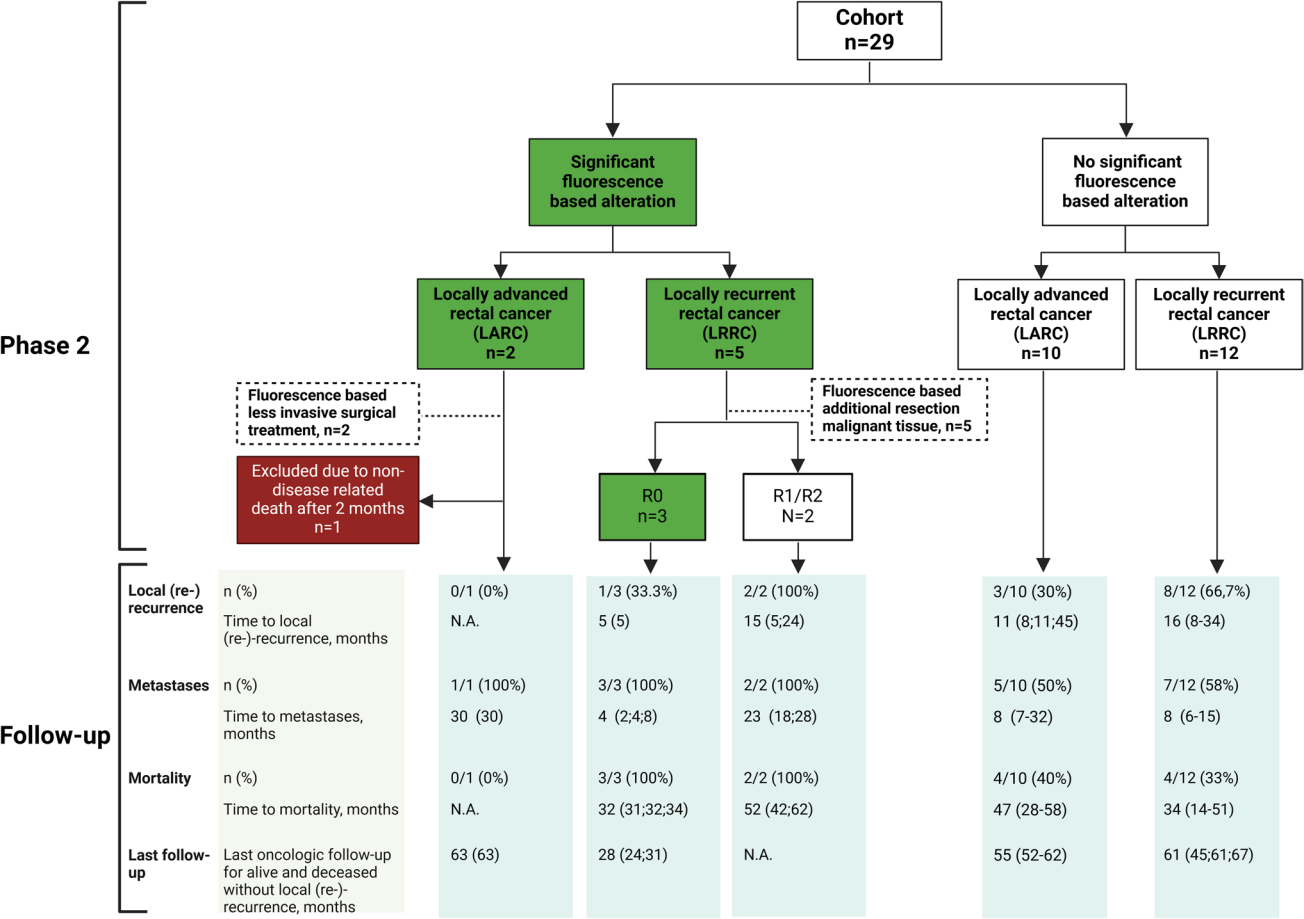
follow-up results stratified based on significant alteration or no significant alteration using near-infrared fluorescence-guided surgery with SGM-101. For continues data with > 3 values the median is presented alongside the corresponding interquartile range (IQR) enclosed within brackets. For continuous data with only 3 values or less, the median is presented alongside the corresponding values enclosed within brackets

In 7/29 patients, there was a significant alteration in surgical plan due to NIRF (LARC *n = *2 and LRRC *n = *5). Follow-up results are shown below accordingly and are summarized in Fig. 3.

### Follow-up Patients with Significant Alteration in Surgical Plan

#### LARC (*n = *2)

Of the two patients that had less invasive surgical treatment due to SGM-101, one patient, who did not undergo IORT on the sciatic nerve and turned out to have a pathological complete response, passed away two months after surgery due to an event unrelated to the disease. Consequently, no follow-up data could be obtained for this patient. The second patient, in which tissue around the lateral piriformis could be preserved with subsequently sparing of the internal iliac artery and vein (R0 at pathology), was locally recurrence-free at the last follow-up 63 months post-surgery. This patient was diagnosed with a colorectal lung metastasis 30 months after surgery which was curatively resected. See Fig. 3 for further details.

#### LRRC (*n = *5)

Of the three patients that underwent an R0 resection instead of R1 due to NIRF-guided resection of additional malignant tissue, one patient (33%) developed a unifocal local re-recurrence in the pelvis five months after surgery. The other two patients (67%) did not develop a local re-recurrence until they deceased 31 and 32 months after surgery, respectively, due to disseminated disease. Their last oncological assessments were conducted at 24 and 31 months, respectively. The two patients with a NIRF-guided additional resection that still resulted in an R1 resection, developed a local re-recurrence at 4 and 23 months, respectively. Moreover, they developed distant metastases at 18 and 28 months and deceased 42 and 62 months after surgery, respectively. Additional details are summarized in Fig. 3.

### Follow-up Patients Without Significant Alteration in Surgical Plan

#### LARC (*n = *10)

Eight patients had no alteration of the surgical plan and two patients had a minor, additional, false positive (fluorescent but benign) resection based on NIRF (all patients R0). Three patients (30%) developed a local recurrence (8, 11 and 45 months, respectively), five (50%) patients developed distant metastases after a median of 8 months (IQR: 7–32) and four patients (40%) deceased after a median of 47 months (IQR 28–58).

#### LRRC (*n = *12)

In total 12 patients with LRRC had no significant alteration of the surgical plan (10/12 (83%) R0). In two of these patients a false positive (fluorescent but benign) additional resection was performed of remaining fluorescence in the wound bed. These resections were all minor in size and did not lead to post-operative morbidity higher than expected. Eight patients (67%) developed a local re-recurrence (R0 75%) after a median of 16 months (IQR: 8–34) and 7 patients (58%) developed distant metastases after a median of 8 months (IQR: 6–15), as shown in Fig. 3. Four patients (33%) deceased after a median of 34 months (IQR: 14–51).

### Balance of Potential Drawbacks and Benefits SGM-101

**Fig. 4:**
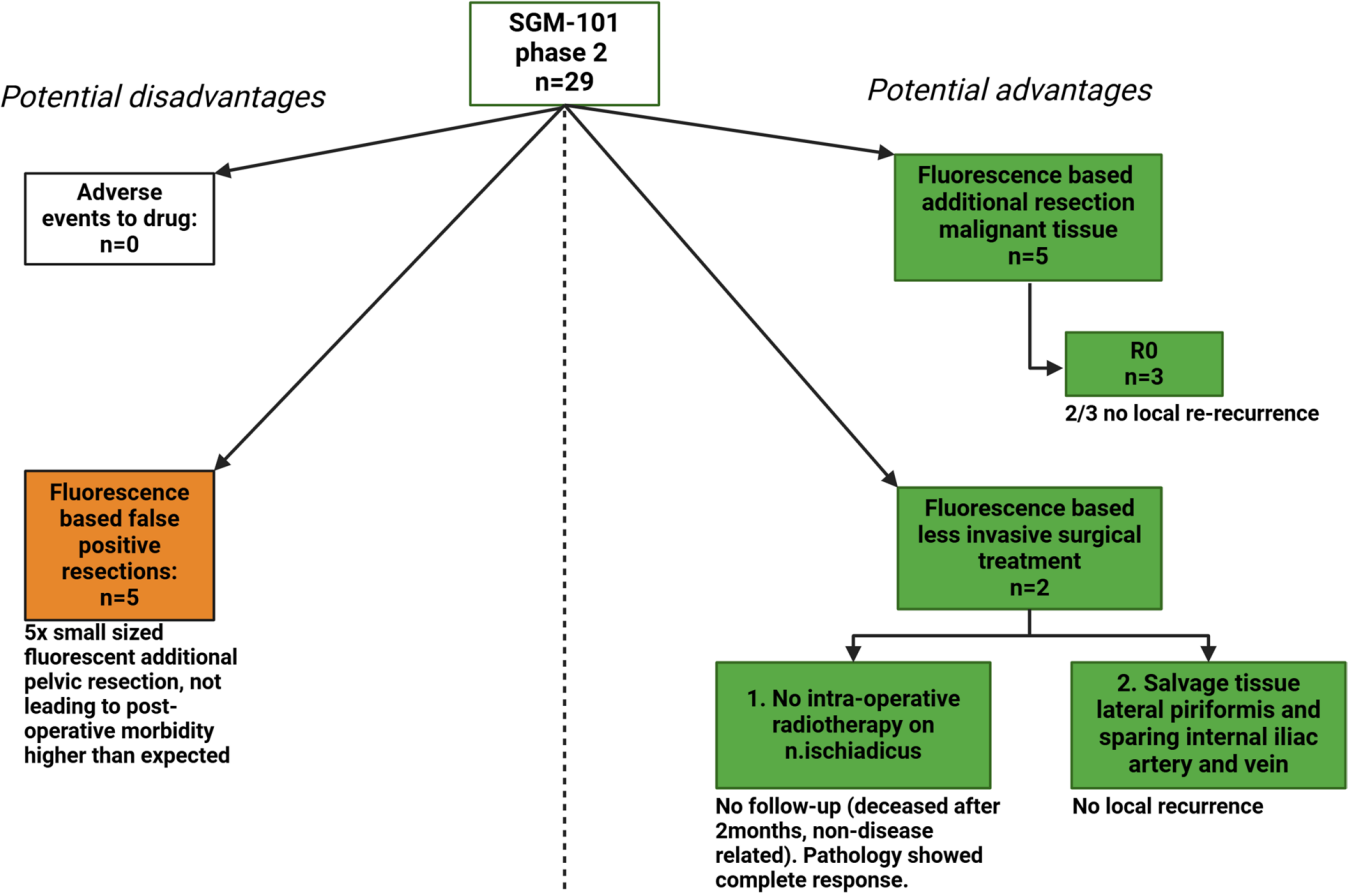
Balancing the clinical disadvantages of the usage of SGM-101 (left side) and the potential advantages (right side)

Figure 4 illustrates the potential advantages and disadvantages of SGM-101. There were no reported adverse events or side effects related to the infusion of SGM-101. However, in five patients, false positive additional resections were performed (fluorescent but benign tissue). These resections were all minor in size and did not lead to post-operative morbidity higher than expected levels. One of the false positive NIRF-based minor additional resections occurred in patient 4 (Supplementary Table 1), in whom also two true positive (fluorescent and malignant tissue) additional resections were performed.

Concerning the potential benefits of SGM-101: in five patients the imaging agent accurately identified additional true positive tissue, resulting in R0 resections in three patients, of whom two patients remained free from local (re-)recurrence in the follow-up. In two patients less invasive surgery was performed. One of these patients, with a pathological complete response, deceased non-disease related two months after surgery. The other patient did not develop a local recurrence and is still alive.

## Discussion

During the NIRF-guided phase 2 trial in LARC and LRRC patients [11, 12], SGM-101 resulted in additional resections of malignant tissues in a subset of patients while enabling less invasive surgery in others. While the use of SGM-101 led to improved intra-operative surgical outcomes, we could not prove long-term oncological benefit for the whole cohort. However, the subset of patients whose surgical approach was rightfully modified based on NIRF – either by performing less invasive surgery or extending the surgical resection—showed favorable long-term outcomes.

There is an increasing number of trials evaluating tumor-targeted fluorescence imaging. Up to our knowledge, none of these trials have evaluated long-term impact so far. This could be attributed to the fact that the majority of these trials were conducted relatively recently. Nevertheless, investigating long-term patient outcome is crucial in determining the potential benefit of NIRF-guided surgery. For this reason, the SGM-LARRC-trial (NCT04642924), currently enrolling a total of 203 patients with LARC or LRRC, is powered to assess both intra-operative benefit as long-term outcomes. Unfortunately, the long-term results of this trial are not expected soon. Therefore, despite its limiting sample size, this is a first study to evaluate long-term impact of intra-operative tumor-targeted fluorescence imaging.

Similar to the objective of the current trial, in recent decades, new techniques and treatment regimens have been evaluated to enhance local tumor control in patients with rectal cancer. The Dutch TME trial showed that the addition of neoadjuvant radiotherapy in rectal cancer patients had a positive impact on local recurrence rate [16]. Similarly, the German CAO/ARO/AIO-94-trial showed that neoadjuvant instead of adjuvant chemoradiotherapy, decreased the local recurrence rate [17]. Additionally, intraoperative radiotherapy (IORT) may have an effect to enhance local tumor control [18]. Within our relatively small cohort, the application of NIRF imaging resulted in the identification of additional malignant tissue in five patients, thereby achieving an R0 instead of an R1/R2 in three patients. During follow-up of these three patients a re-recurrence rate of 33% (1 out of 3 patients) was observed. If these patients had remained R1, the anticipated re-recurrence rate, based on historical cohorts, were likely to approach 70–100% (15). Hence, despite the limited number of patients, a positive effect on local control for these patients is suggested. Unfortunately, this trend could not be observed for the entire cohort: a 5-year local (re-)recurrence rate of 65% for LRRC patients and 25% for LARC patients was observed. These numbers are slightly higher than historical cohorts, reporting 49–62% and 7%−23.5% local (re-)recurrence rates at 5-year follow-up, respectively [17, 19, 20]. Despite the possibility of a small sample size influence, it might be explained by the surgeries taking place in specialized last resort rectal cancer surgery centers. Overall 5-year survival rates for this cohort were 53% for LRRC patients and 50% for LARC patients, reflecting historical cohorts with 5-year survival rates of 31% – 41% for LRRC patients and 52%−66% for LARC patients [2, 3, 6, 21–24].

To further assess the potential value of NIRF-guided surgery with SGM-101 in rectal cancer, an overview of the potential drawbacks and benefits are illustrated in Fig. 4**.** Although SGM-101 infusion did not lead to adverse events, a potential drawback was observed in five patients where false positive (fluorescent but benign tissue) additional resections were performed. All excisions were small sized and did not lead to post-operative morbidity exceeding expected levels. On the other hand, in five patients, true positive (fluorescent and malignant) tissue was removed solely based on the fluorescence signal of SGM-101, resulting in an R0 resection in three patients, two of whom remained local re-recurrence free. In two patients NIRF-guided surgery resulted in significant less invasive surgical treatment and still were R0. Unfortunately, one of these patients, in whom IORT on the sciatic nerve was omitted, passed away two months after surgery due to an event unrelated to the disease. However, this patient had a pathological complete response, indicating that SGM-101 accurately identified the justification for a surgical downgrade. The other patient is still alive and did not develop a local recurrence. Given that the potential drawbacks are minor in comparison to the potential benefits in achieving complete tumor removal, the overall impact is considered positive.

## Conclusion

This is the first study reporting on follow-up data of patients that underwent surgery with the guidance of tumor-targeted near-infrared fluorescence. Although SGM-101 resulted in warranted changes in surgical management intra-operatively, no improved long-term benefit could be observed for the entire cohort. However, the specific subset of patients in whom surgical management was modified based on NIRF imaging—either through downstaging of the surgical treatment or the resection of additional malignant tissue that would have otherwise been missed—demonstrated favorable long-term outcomes*.* Moreover, when balancing the potential disadvantages and advantages of SGM-101 FGS, the net effect is considered positive. Given the small sample size, results of the larger ongoing multicenter trials (SGM-clin03 (NCT03659448) and SGM-LARRC (NCT04642924)) are awaited. The data associated with this study are presented in the paper.

## Supplementary Information

Below is the link to the electronic supplementary material.

Supplementary file1 (DOCX 17 KB)

## Data Availability

The data associated with this study are presented in the paper. Additional imaging data from this study are available upon request to the Corresponding Author.
