# Supplementary material for: Long-term Local Control Following CEA-targeted Fluorescence-guided Surgery in Patients With Locally Advanced and Recurrent Rectal Cancer
Source: Mol Imaging Biol. 2025 Jun 5;27(4):629–37. doi: 10.1007/s11307-025-02021-4 (PMC12405016; doi:10.1007/s11307-025-02021-4)
Supplement: Supplementary file 1 — Supplementary file1 (DOCX 17 KB) [file 11307_2025_2021_MOESM1_ESM.docx]

**Electronic Supplementary Material**

**Long-term local control following CEA-targeted fluorescence-guided surgery in patients with locally advanced and recurrent rectal cancer

Journal: Molecular Imaging and Biology**

Mats I. Warmerdam M.D.^1^, Davy M.J. Creemers M.D.^2,3^, Miranda Kusters M.D., Ph.D.^4^, Koen C.M.J. Peeters M.D., Ph.D.^1^, Fabian A. Holman M.D., Ph.D.^1^, J. Sven D. Mieog M.D., Ph.D.^1^_,_ Francoise Cailler Ph.D.^5^, Pim J.W.A. Burger M.D., Ph.D.^2^, Jacobus Burggraaf M.D., Ph.D.^6^, Harm J.T. Rutten M.D., Ph.D.^2,3^, Cornelis Verhoef M.D., Ph.D.^7^, Alexander L. Vahrmeijer M.D., Ph.D.^1^, Denise E. Hilling M.D., Ph.D.^1,7^

1. Department of Surgery, Leiden University Medical Center, Leiden, The Netherlands
2. Department of Surgery, Catharina Hospital Eindhoven, Eindhoven, The Netherlands
3. Department of GROW, School for Developmental Biology & Oncology, Maastricht University, Maastricht, the Netherlands
4. Department of Surgery, Amsterdam University Medical Center (AUMC), Amsterdam, The Netherlands
5. Surgimab, Montpellier, France
6. Center for Human Drug Research (CHDR), Leiden, The Netherlands
7. Department of Surgical Oncology and Gastrointestinal Surgery, Erasmus MC Cancer Institute, Rotterdam University Medical Center, Rotterdam, The Netherlands

**Corresponding author:**
Mats I. Warmerdam, M.D.
Albinusdreef 2,
2333ZA Leiden,
the Netherlands,
Tel: +31715298420.
E-mail: m.i.warmerdam@lumc.nl.

**Introduction**

**Table 1 supplementary:** Per patient overview of surgical plan alterations based on SGM-101 fluorescence-guided surgery as part of the SGM-phase 2 trial.

| Overview surgical plan alterations per patient. | | | | | |
| --- | --- | --- | --- | --- | --- |
|  | **Type of tumor** | **Type of alteration** | **Conclusion** | **Alteration explanation** | **Completeness of resection** |
| 1 | LRRC | additional resection | True positive | Clinically non suspect fluorescent lesion near the left ureter; resected per decision of the surgeon (see **fig.1**) | R0 |
| 2 | LRRC | additional resection | True positive | Clinically non suspect fluorescent lesion proximal of recurrence; resected per decision of the surgeon | R0 |
| 3 | LRRC | additional resection | True positive | Remaining fluorescence in surgical field (clinically non suspect); two reresections performed | R0 |
| 4 | LRRC | 3x additional resection | - 2x True positive (1,2)  - 1x false positive (3) | (1) Remaining fluorescence visible in surgical field (clinically non suspect); re-resection performed. (2) Clinically non suspect fluorescent lesion on lateral pelvic wall; re-resection performed  (3) Clinically non suspect fluorescent lesion on lateral pelvic wall; re-resection performed | R1 |
| 5 | LRRC | additional resection | True positive | Clinically non-suspect fluorescent lesion on lateral pelvic wall; resected per decision of the surgeon | R1 |
| 6 | LRRC | additional resection | False positive | Remaining fluorescence visible in surgical field (clinically non suspect); re-resection presacral and dorsolateral performed | R0 |
| 7 | LRRC | additional resection | False positive | Remaining fluorescence visible in surgical field (clinically non suspect); re-resection performed | R0 |
| 8 | LARC | Additional resection | False positive | Clinically non suspect fluorescent lesion on the bladder wall, resected per decision of the surgeon | R0 |
| 9 | LARC | Additional resection | False positive | Clinically non suspect left fluorescent seminal vesicle left, resected per decision of the surgeon | R0 |
| 10 | LARC | less invasive surgical treatment | True negative | No remaining fluorescence visible in surgical field after resection. Initial surgical plan included a more extensive resection; however, due to negative fluorescence (confirmed benign with FS), the surgeon decided to salvage tissue around the lateral piriformis and the internal arteries and veins. | R0 |
| 11 | LARC | Less invasive surgical treatment | N.A.^1^ | No remaining fluorescence visible in surgical field or specimen after resection. The surgeon assessed the resection as R0, which was confirmed with FS (surgical field and specimen). The surgeon decided to spare the patient the planned IORT, because this would have been on the sciatic nerve, giving morbidity | R0 |

LARC=locally advanced rectal cancer; LRRC=locally recurrent rectal cancer; IORT=intraoperative radiotherapy, R0=margins negative for tumor cells; R1=margins positive for tumor cells; N.A.=not applicable.
^1^Not applicable; patient died two months after surgery (non-disease related)
